# Decreased mitochondrial respiration associates with frailty in community-dwelling older adults

**DOI:** 10.3389/fcell.2024.1301433

**Published:** 2024-05-07

**Authors:** Gianella Liabeuf, Rodrigo Saguez, Carlos Márquez, Bárbara Angel, Roberto Bravo-Sagua, Cecilia Albala

**Affiliations:** ^1^ Instituto de Nutrición y Tecnología de los Alimentos (INTA), Universidad de Chile, Santiago, Chile; ^2^ Escuela de Nutrición y Dietética, Facultad de Salud y Ciencias Sociales, Universidad de las Américas, Santiago, Chile; ^3^ Escuela de Nutrición y Dietética, Facultad de Ciencias de la Salud, Universidad Bernardo O’Higgins, Santiago, Chile; ^4^ Centro Interuniversitario de Envejecimiento Saludable RED21993, Santiago, Chile; ^5^ Advanced Center for Chronic Diseases (ACCDiS), Facultad de Ciencias Químicas y Farmacéuticas y Facultad de Medicina, Universidad de Chile, Santiago, Chile

**Keywords:** mitochondria, respiration, frailty, PBMC, aging

## Abstract

Aging population has led to an increased prevalence of chronic and degenerative pathologies. A manifestation of unhealthy aging is frailty, a geriatric syndrome that implies a non-specific state of greater vulnerability. Currently, methods for frailty diagnosis are based exclusively on clinical observation. The aim of this study is to determine whether the bioenergetic capacity defined as mitochondrial oxygen consumption rate (OCR) of peripheral circulation mononuclear cells (PBMC) associates with the frailty phenotype in older adults and with their nutritional status. This is a cross-sectional analytic study of 58 participants 70 years and older, 18 frail and 40 non-frail adults, from the ALEXANDROS cohort study, previously described. Participants were characterized through sociodemographic and anthropometric assessments. Frail individuals displayed a higher frequency of osteoporosis and depression. The mean age of the participants was 80.2 ± 5.2 years, similar in both groups of men and women. Regarding the nutritional status defined as the body mass index, most non-frail individuals were normal or overweight, while frail participants were mostly overweight or obese. We observed that OCR was significantly decreased in frail men (*p* < 0.01). Age was also associated with significant differences in oxygen consumption in frail patients, with lower oxygen consumption being observed in those over 80 years of age. Therefore, the use of PBMC can result in an accessible fingerprint that may identify initial stages of frailty in a minimally invasive way.

## Introduction

Worldwide population aging and the consequent epidemiological change has led to an increased prevalence of chronic and degenerative pathologies (Guo et al., 2022). This associates with a progressive decline in tissue functions, which reduces the organism’s capacity to maintain homeostasis and decreases its adaptation to internal and external stresses (Fedarko, 2011; López-Otín et al., 2013). At the organismal level, this translates into frailty, a geriatric syndrome that implies a non-specific state of greater vulnerability as a consequence of multisystem dysregulation, which entails an accelerated decrease in physiological reserves (Cesari et al., 2017). Frailty is a manifestation of unhealthy aging, and the risk of suffering from it worsens in the presence of acute or chronic diseases. Likewise, the exposure of frail older people to some stressor makes them more prone to hospitalizations, dependency, institutionalization and death (Clegg et al., 2013; Albala et al., 2017; Ofori-Asenso et al., 2019).

Globally, the incidence of frailty and pre-frailty has been estimated at 43.4 and 150.6 new cases per 1,000 person-years, respectively. Incidence rates for frailty and pre-frailty vary by sex, diagnostic criteria, and country income level (Ofori-Asenso et al., 2019). In Chile, the ALEXANDROS cohort study showed a prevalence of frailty of 13.9% (Albala et al., 2017) and an incidence of frailty of 40/1,000 person-years of observation (Albala et al., 2020).

Within the operational definitions of frailty, the most widely used is the one that defines a Physical Frailty Phenotype proposed by Linda Fried, which includes five physical parameters: weakness, involuntary weight loss, tiredness, decreased strength, and low physical activity. An individual is considered frail if they meet three or more of the criteria, pre-frail if they meet one or two, and non-frail (also called robust) if none of them are met (Fried et al., 2001; Fried, 2016).

Several studies show a strong association between frailty and malnutrition older adults, where both conditions share common risk factors, such as weight loss, functional decline, and worsening of other pre-existing clinical conditions (Falsarella et al., 2015; Abizanda et al., 2016; Ferriolli et al., 2017; Xu et al., 2020). Low weight and a greater waist circumference have been found to increase the risk of frailty, while being overweight is a protective factor (Ferriolli et al., 2017). In relation to body composition, frail older adults present a decrease in muscle mass, low bone mass, and a higher fat percentage (Falsarella et al., 2015; Xu et al., 2020).

On the other hand, the alteration of energy metabolism, particularly at the mitochondrial level, is inherent to aging, having observed that frail older adults have a lower basal metabolic rate (Abizanda et al., 2016). At the cellular level, an aged and imperfect mitochondrial network may contribute to age-associated metabolic dysfunction (Campisi et al., 2011). Studies have reported that mitochondrial respiration in peripheral circulation mononuclear cells (PBMC) serves as an indicator of general energy metabolism, and associates with chronic and degenerative pathologies in old age such as diabetes or Parkinson’s disease (Busquets-Cortés et al., 2018).

At rest, PBMC depend mainly on mitochondrial respiration to adapt to metabolic demand, and it has been suggested that their bioenergetic profile reflects the mitochondrial function of other tissues, and that it relates to the degree and prognosis of disease (Riou et al., 2020). Metabolic function assessment of PBMC for disease treatment and diagnosis is a relatively recent area of research (Wagner et al., 2016).

Currently, methods for frailty diagnosis are based exclusively on clinical observation (Sepúlveda et al., 2022), thus the measurement of the respiratory capacity of PBMC could reflect functional decline and serve as a biomarker. Therefore, the use of PBMC can result in an accessible fingerprint of degenerative processes that may allow for the identification of initial stages of functional decline associated with frailty conditions in older adults in a minimally invasive way.

The aim of this study is to determine whether the bioenergetic capacity of PBMC associates with the frailty phenotype in older adults and with their nutritional status.

## Materials and methods

### Participants and study design

This is a cross-sectional analytic study in 58 (65.5% women) participants from the ALEXANDROS cohort study, previously described. Briefly, ALEXANDROS is a cohort study in community-dwelling adults 60 years and older from Santiago, Chile, designed to study disability conditions associated with the nutritional status in older Chileans (Albala et al., 2011). It started in 2000, with addition of new participants in 2007 and 2012, with successive follow-ups in 2004–2005, 2007-2008, 2010-2012, 2015-2017 and 2020-2021-2022.

For this study, we selected participants from the 2020 evaluation using as inclusion criteria being 70 years or older, and having completed all the assessments mentioned in the subsections below (i.e., geriatric assessment, short physical performance battery, frailty assessment, blood extraction and oxygen consumption rate). From a total of 87 participants complying with these criteria, 18 individuals were frail (20.7%), all of which were included in this study. As control group, 40 non-frail older adults were randomly selected from the rest of the participants, with a comparable composition in terms of age and sex.

### Geriatric assessment

Geriatric assessment included self-reported falls, osteoporosis, cancer, multimorbidity, and perception of health. A score 5 or higher was considered for the diagnosis of depression according to the Geriatric Depression Scale (GDS-15) (Yesavage and Sheikh, 1986). Quality of life was assessed by applying a single question “In general, how would you say your quality of life is currently?”, previously validated in Chile in a representative sample of older adults, using the SF-36 as gold standard (Lera et al., 2013). To evaluate subjective wellbeing, the Life Satisfaction Index was used as described by Neugarten and others (Neugarten et al., 1961).

### Short physical performance battery (SPPB)

SPPB comprised three evaluations (Pritchard et al., 2017). First, a balance test consisting in the capacity to stand for 10 s with feet side-by-side (1 point), in semi-tandem (1 point) and in tandem (2 points). Second, a squat test measuring the time to complete five stand-ups and sit-downs from a chair (squat time <11.2 s scores a maximum of four points). Finally, a speed evaluation measuring the time to walk 3 m (walking time <4.82 s scores a maximum of four points). Thus, the maximum score of the SPPB test is 12.

### Frailty assessment

Frailty was identified as meeting at least three out of five criteria: self-reported unintentional loss of 5 kg or more in the last 6 months, self-report of fatigue or feeling exhausted, self-report of difficulty for walking eight blocks, walking speed less than 0.8 m/s (measured as part of the SPPB), and weakness by dynamometry ≤27 kg in men and ≤15 kg in women according to reference values of handgrip strength for Chilean older adults (Lera et al., 2018). These are based on Fried’s frailty definition, adapted to the Chilean population, as we previously described (Albala et al., 2017): we used walking difficulty as a proxy of low physical activity.

### Blood extraction and PBMC isolation

Prior to the physical assessment, blood samples were drawn after a fast of 8–10 h, between 9.00 and 10.00 a.m., through venipuncture of the arm. Samples were collected in two Vacutainer Cell Preparation Tubes (CPT) with sodium heparin as anticoagulant (16 mL in total). CPT are pre-filled with Ficoll-Hypaque, which forms a density gradient upon centrifugation that sediments erythrocytes and granulocytes to the bottom of the tube. Additionally, CPT contain a gel plug whose buoyancy positions it just above the aforementioned cells, separating them from PBMC, which settle just above the plug (Puleo et al., 2017). After the blood extraction, CPT were immediately centrifuged for 20 min at 1,500 rcf at room temperature. Then, 2 mL of cell suspension were extracted from the gradient just above the plug, and were further purified through two consecutive washes with PBS (10 mL) and centrifugation at 300 rcf for 15 min at room temperature. Subsequently, cells were resuspended in 500 µL of DMEM 10% fetal bovine serum and counted using a BioRad TC20 cell counter according to the manufacturer’s specifications. Mean cell viability was 53% ± 21% (standard deviation).

### Oxygen consumption rate

Immediately after isolation, 2 × 10^6^ viable PBMC were placed in the chamber of a Clark Oxygraph Plus oxygraph (Hansatech Instruments, Norfolk, United Kingdom) in a total volume of 350 µL of DMEM 10% fetal bovine serum at 37°C. The equipment measures real-time oxygen concentration within the chamber. After placing the cells and a brief stabilization period, a steady decrease in the oxygen concentration takes place, reflecting mitochondrial respiration. Oxygen consumption rate (OCR) was measured for 2 min, and the basal respiratory rate was calculated as the rate of the decrease in oxygen concentration in the chamber per minute (nmol/mL/min). See Supplementary Figure S1 for a representative tracing of OCR.

### Ethics

All procedures were carried out in accordance with the latest version of the Declaration of Helsinki. The research protocol was approved by the Ethics Committee of the Institute of Nutrition and Food Technology (INTA) of the University of Chile, Santiago (project code P15-2018, approved on 12 September 2018). All participants signed an informed consent prior to entering the study.

### Statistical analysis

Results are expressed as mean ± standard deviation and 95% CI for continuous variables. For categorical variables, the results were expressed in percentages and number of subjects (n). To verify the normality of the data, the Kolmogorov-Smirnov test was used. Statistical comparisons between frail and non-frail were performed using the Student’s t-test when the variables presented a normal distribution, and the non-parametric Mann-Whitney U test for variables without normal distribution. The Chi square test (χ^2^) was applied to estimate the association between categorical variables.

Logistic regression models were performed to analyze the association between frailty and OCR adjusting for other variables to rule out confounding effects. For model 1, we adjusted for sex and age groups (70-74, 75-78 and ≥80 years). Model two assessed the same variables plus body composition (lean mass/fat mass ratio). Model three considered the aforementioned plus osteoporosis and multimorbidity, and model four included the nutritional state grouped according to body mass index (underweight <23, normal 23-27.9, overweight 28-31.9 and obese ≥32 kg/m^2^, according to Chilean Ministry of Health guidelines). Aside from OCR and lean mass/fat mass ratio, all variables were considered as categorical. The Hosmer-Lemeshow test was used to check if each of the proposed models can explain the observed data.

Statistical significance for all tests was set at *p* < 0.05. All statistical analyses were performed with STATA 16.1 software (StataCorp LLC, College Station, TX).

## Results

### Sample description

The sociodemographic and health characteristics of the sample are shown in Table 1, according to frailty. The average age of the participants was 80.2 ± 5.2 years, similar in non-frail and frail, both for men and women. The overall frequency of sarcopenia and falls reached 26.1% and 31%, respectively, with no differences by sex or frailty status. Osteoporosis was higher in frail people (6.1% vs. 35.7%, *p* < 0.05). A similar situation was observed for depression (12.5% vs. 44.4%, *p* < 0.05) and average/poor quality of life (10% vs. 38.9%, *p* < 0.05). Regarding multimorbidity, 74.1% of all the participants had two or more diseases, with no significant differences between non-frail and frail.

**TABLE 1:** Sociodemographic and health characteristics according to frailty.

Variables	Non-frail (n = 40)	Frail (n = 18)	Total (n = 58)
Sex % (n)			
Women	68.4 (26)	31.6 (12)	65.5 (38)
Men	70.0 (14)	30.0 (6)	34.5 (20)
Age (years)			
Mean ± SD	79.9 ± 5.4	80.8 ± 5.7	80.2 ± 5.2
95% CI	78.3–81.5	77.9–83.7	78.8–81.6
Schooling (years)			
Mean ± SD	9.1 ± 3.6	6.3 ± 5.1	8.5 ± 3.9
IQR	7.3–10.8	1.8–14.3	6.9–10.3
Falls % (n)	30.0 (12)	33.3 (6)	31.0 (18)
Osteoporosis % (n)*	6.1 (2)	35.7 (5)	14.9 (7)
Sarcopenia % (n)	21.9 (7)	35.7 (5)	26.1 (12)
Depression % (n)*			
GDS-15 ≥ 5	12.5 (5)	44.4 (8)	22.4 (13)
Cancer % (n)	15.0 (6)	-	10.3 (6)
Multimorbidity % (n)			
<2 diseases	32.5 (13)	11.1 (2)	25.9 (15)
≥2 diseases	67.5 (27)	88.9 (16)	74.1 (43)
Current quality of life % (n)			
Excellent/good	90.0 (36)	61.1 (11)	81.0 (47)
Average/bad*	10.0 (4)	38.9 (7)	19.0 (11)
Self-perception of health % (n)	82.5 (33)	66.7 (12)	77.6 (45)
Excellent/good	17.5 (7)	33.3 (6)	22.4 (13)
Average/bad			
Life Satisfaction Index median (n)	12 (2)	12 (2)	12 (3)

*p < 0.05, **p < 0.01 according to the χ^2^ test.

SD, standard deviation; CI, confidence interval; GDS-15, Geriatric Depression Scale; IQR, interquartile range.

The nutritional status (grouped according to body mass index) and physical performance data are shown in Table 2, expressed as percentage and as mean ± SD. Regarding the nutritional status, most non-frail individuals were normal or overweight, while frail participants were mostly overweight or obese; however, there were no differences between the groups. Overall, women showed less force than men in the dynamometry assessment, as expected. In non-frail men, the mean force exerted was significantly higher than in frail men (*p* < 0.01); meanwhile, there were no differences between both groups of women in terms of dynamometry. Non-frail individuals displayed faster squat time compared to the frail (*p* < 0.05). Walking speed was non-significant between groups.

**TABLE 2:** Nutritional status and physical performance in frail and non-frail older adults.

Variables	Non-frail (n = 40)	Frail (n = 18)	Total (n = 58)
Nutritional status % (n)			
<23 kg/m^2^	12.5 (5)	11.1 (2)	12.1 (7)
23–27.9 kg/m^2^	40.0 (16)	16.7 (3)	32.8 (19)
28–31.9 kg/m^2^	35.0 (14)	44.4 (8)	37.9 (22)
≥32 kg/m^2^	12.5 (5)	27.8 (5)	17.2 (10)
Dynamometry (kg)			
Mean ± SD			
Men**	31.0 ± 4.6	22.3 ± 7.9	28.4 ± 6.9
Women	16.5 ± 4.5	14.0 ± 2.7	15.7 ± 4.1
<27 kg | <15 kg % (n)**	22.5 (9)	83.3 (15)	41.4 (24)
Squat time (s)			
Mean ± SD*	9.3 ± 2.1	11.0 ± 3.2	9.8 ± 2.6
≥11.2 s % (n)*	12.5 (5)	38.9 (7)	20.7 (12)
Walking speed (s)	4.2 ± 1.2	4.4 ± 1.4	4.3 ± 1.2
Mean ± SD	97.5 (39)	100 (18)	98.3 (57)
≥0.8 m/s % (n)			
Balance subscale median (p25-p75)	4 (3-4)	3.5 (3-4)	4 (3-4)
Total SPPB score median (p25-p75)	12 (10-12)	11 (10-12)	11 (10-12)

Differences were assessed via χ2 test (nutritional status), Student’s t-test (dynamometry, squat time and walking speed), or Mann-Whitney’s test (balance subscale and total SPPB, score). *p < 0.05, **p < 0.01.

SPPB, short physical performance battery; SD, standard deviation. p25-p75, interquartile range.

### Respirometry

Table 3 shows the OCR (nmol/mL/min) of PBMC of controls and frail individuals, distributed according to sex and age, and expressed as mean ± SD. We observed that OCR was significantly decreased in frail men (*p* < 0.01). Age was also associated with significant differences in oxygen consumption in frail patients, with lower oxygen consumption being observed in those over 80 years of age.

**TABLE 3:** Frailty and oxygen consumption according to sex and age.

Variables	Oxygen consumption rate (nmol/mL/min)					
	Non-frail (n = 40)		Frail (n = 18)		Total (n = 58)	
	mean ± SD	(n)	mean ± SD	(n)	mean ± SD	(n)
Sex						
Women	1.6 ± 1.5	(26)	1.2 ± 1.6	(12)	1.5 ± 1.5	(38)
Men**	1.6 ± 0.9	(14)	0.34 ± 0.2	(6)	1.2 ± 0.9	(20)
Age						
70–74 years **	2.6 ± 2.1	(7)	0.3 ± 0.4	(2)	2.1 ± 2.0	(9)
75–79 years	1.1 ± 0.8	(13)	1.5 ± 1.8	(8)	1.3 ± 1.3	(21)
≥80 years **	1.6 ± 1.1	(20)	0.4 ± 0.3	(8)	1.2 ± 1.1	(28)

**p < 0.01 according to Mann-Whitney’s *U* test. SD, standard deviation.

**FIGURE 1:**
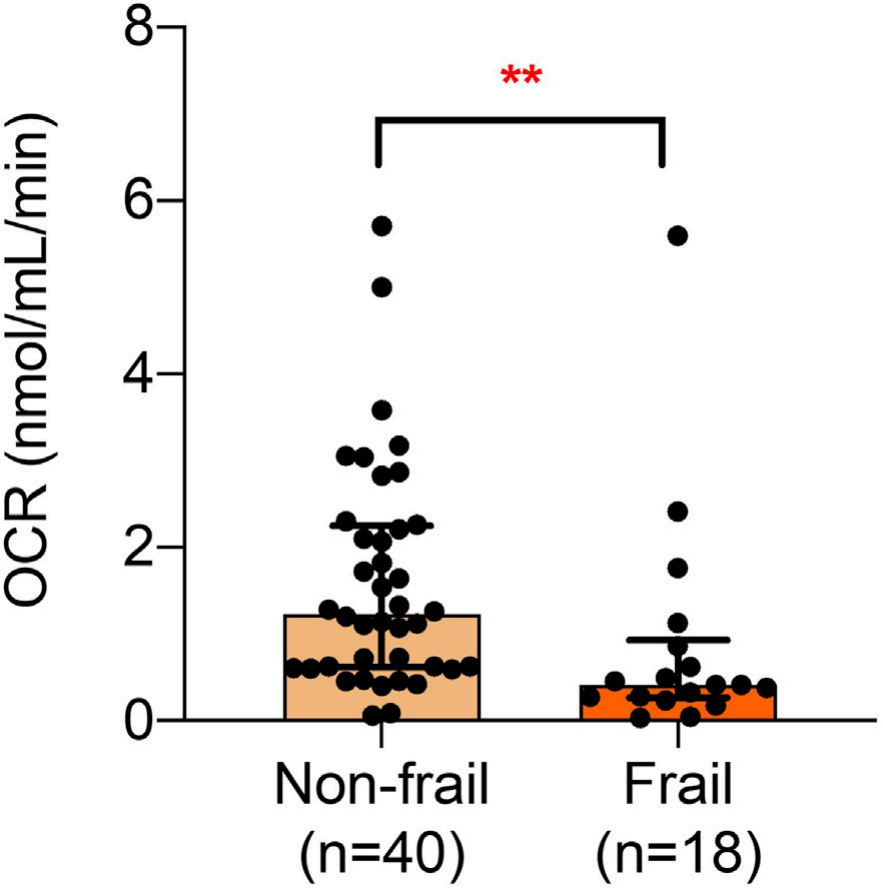
OCR of frail and non-frail older adults. A significant difference is observed in the median oxygen consumption according to frailty.

Figure 1 shows the overall OCR of PBMC of non-frail and frail older adults. Frailty was found to significantly affect PBMC OCR.

### Respirometry and frailty association

Table 4 shows the logistic regression analysis for frailty and OCR adjusted for sex and age (model 1), lean mass/fat mass ratio (model 2), osteoporosis, multimorbidity (model 3) and nutritional status (model 4). We observed a negative association between OCR and frailty in models 2-4.

**TABLE 4:** Logistic regression models for frailty, adjusted for sex, age, and nutritional status.

Variables	Model 1 ‡		Model 2 §		Model 3 ||		Model 4 {	
	OR (95% CI)	p	OR (95% CI)	p	OR (95% CI)	p	OR (95% CI)	p
OCR	0.56 (0.30-1.07)	0.078	0.31 (0.11-0.92)	0.034	0.29 (0.09-0.89)	0.030	0.26 (0.08-0.85)	0.026
Sex (women)	1.13 (0.33-3.91)	0.844	1.02 (0.17-6.14)	0.981	0.72 (0.09-5.66)	0.752	0.94 (0.11-8.32)	0.959
Age groups*								
75–78 years	1.04 (0.12-9.09)	0.975	1.02 (0.09-11.4)	0.990	0.82 (0.05-12.62)	0.885	2.42 (0.10-57.22)	0.585
≥79 years	1.37 (0.22-8.48)	0.732	1.06 (0.14-8.19)	0.958	0.76 (0.084-6.82)	0.803	2.00 (0.14-29.62)	0.614
Lean mass/fat mass ratio			0.92 (0.33-2.62)	0.882	0.80 (0.23-2.80)	0.722	1.53 (0.37-6.37)	0.562
Osteoporosis					13.80 (1.31-145.7)	0.029	7.44 (0.50-111.2)	0.146
Multimorbidity ≥3 diseases					2.17 (0.41-11.54)	0.365	2.72 (0.43-17.17)	0.288
Nutritional state†								
Underweight							2.98 (0.18-48.12)	0.442
Overweight							3.82 (0.29-49.71)	0.306
Obese							15.20 (0.71-323.5)	0.081

OCR: oxygen consumption rate; OR: odds ratio; CI: confidence interval; Reference category for dependent variable: non-frail; *70–74 years group is used as reference group; ^†^ Normal nutritional state is used as reference group; Hosmer-Lemeshow’s goodness-of-fit test: ^‡^ Chi^2^(8) = 7.60; *p* = 0.47; § Chi^2^(8) = 4.16; *p* = 0.84; || Chi^2^(8) = 13.40; *p* = 0.10; { Chi^2^(8) = 9.52; *p* = 0.30.

**FIGURE 2:**
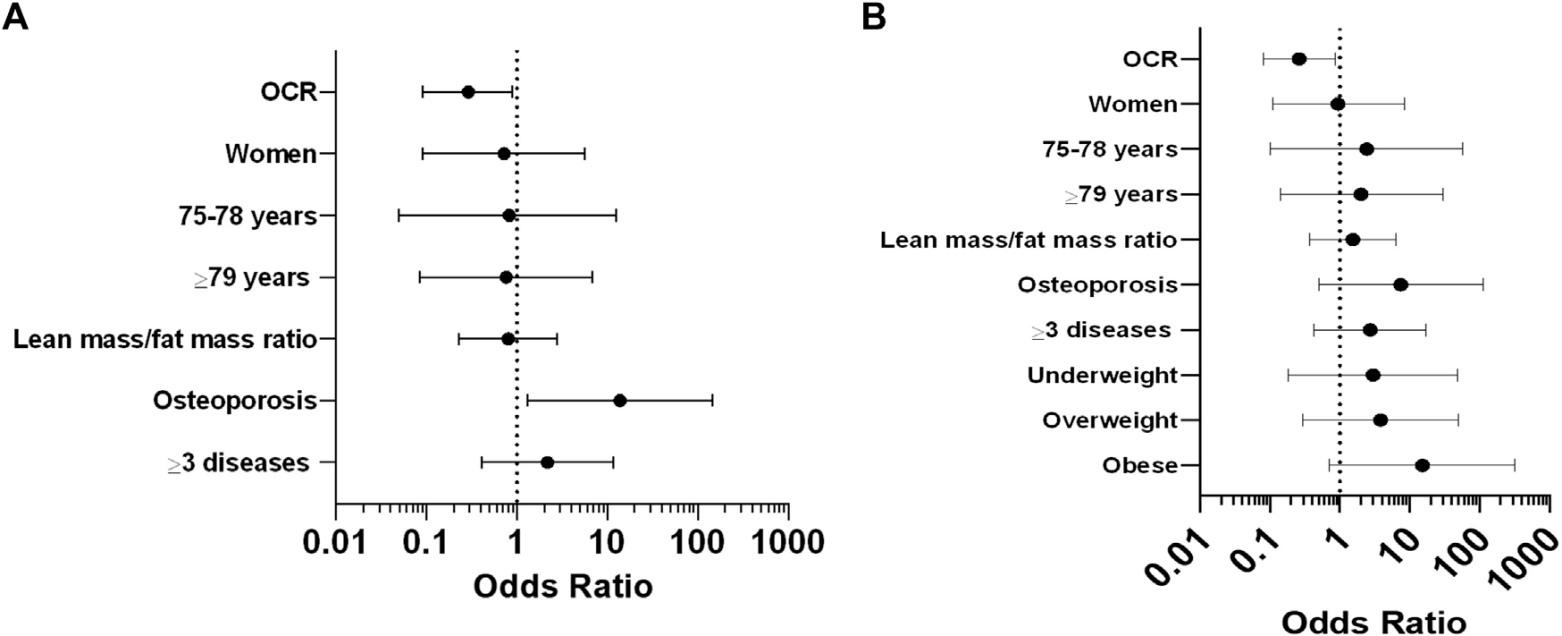
Logistic regression analysis for frailty and OCR. **(A)** Forest Plot for OR of frailty according to OCR adjusted by sex, age, lean/fat mass ratio, osteoporosis and multimorbidity (model 3). **(B)** Forest Plot for OR of frailty according to OCR adjusted by sex, age, lean/fat mass ratio, osteoporosis, multimorbidity and nutritional status (model 4).

The Forest plot for the OR of frailty according to OCR, adjusted by sex, age, lean/fat mass ratio, osteoporosis, multimorbidity (model 3) and nutritional status (model 4), where OCR is negatively associated with frailty is shown in Figure 2 A (model 3) and B (model 4).

## Discussion

In the present study we found a significant association between frailty and decreased PBMC respiration, as the mitochondrial respiration of frail older men was lower than their non-frail peers. Similar results were obtained by Jacob et al. (Jacob et al., 2021), whose analyzes were performed in pre-frail and frail older adults considering nutritional interventions and physical activity. Reportedly, there are sex differences in PBMC mitochondrial function in healthy adults, with men having lower bioenergetics compared to women (Silaidos et al., 2018). In our study, these differences may explain why older women maintain the OCR of their PBMC even in frail condition, contrary to men.

This study showed that decreased respiration occurs early in the natural history of the disease (Jacob et al., 2021). Similarly, Andreaux *et al.* showed decreased mitochondrial function in prefrail subjects compared to non-frail (Andreux et al., 2019), although they measured it in muscle through non-invasive phosphorus magnetic resonance spectroscopy (31P-MRS) and *ex vivo* in muscle explants through the abundancy of mitochondrial complexes proteins and mitochondrial DNA content. In our work, we used a simpler method, minimally invasive to assess frailty. Importantly, other studies suggest that the bioenergetic profile of PBMC reflects mitochondrial function in other tissues (Kramer et al., 2014; Justice et al., 2018).

In neurodegenerative diseases, PBMC from patients with Alzheimer’s disease have been reported to have mitochondrial dysfunction compared to patients with mild cognitive impairment and healthy controls (Delbarba et al., 2016; Mahapatra et al., 2023). Additionally, studies of patients with Parkinson’s disease showed that PBMC had increased mitochondrial dysfunction and oxidative stress (Smith et al., 2018) and in other movement disorders, mitochondrial respiration of PBMC was decreased (Michalak et al., 2017).

Frailty requires early recognition, especially if we consider its relative irreversibility, its high prevalence, the increased health burden, higher mortality, and adverse health outcomes (Lahousse et al., 2014; Álvarez-Bustos et al., 2022). If we consider that the diagnosis is not always timely and that it is also focused on physical frailty, the interventions offer limited strategies that are usually aimed at improving muscle mass and are used rather in late stages (Troncoso-Pantoja et al., 2020). Early diagnosis may help decrease the risk of pre-frailty transitioning to frailty, especially through the prescription of resistance-, balance- and functional-based exercise as well as adequate intake of calories, protein and vitamin D supplementation (Woolford et al., 2020). The use of less invasive biomarkers such as PBMC offer an opportunity to predict frailty risk as they may reflect changes at the systemic level. In this context, the strength of this study is that it proposes the use of a biomarker that complements the clinical assessment of frailty. Future longitudinal studies should assess the prognostic value of OCR to anticipate whether a non-frail person is at risk of development frailty, to implement timely interventions.

One of the limitations of this study was the insufficient sample size that did not allow us to demonstrate the association between nutritional status and OCR in frail older adults. If we consider the role of nutrition in aging, the changes associated with it cause a decrease in the intake and absorption of nutrients, increasing the risk of malnutrition and the appearance of chronic conditions that favor functional decline (Verlaan et al., 2017). The existing relationship between frailty and nutrition is characterized by decreased energy metabolism, deterioration of skeletal muscle mass and quality, hormonal changes, and inflammation. Several studies show a strong association between frailty and malnutrition in older adults, contributing to greater vulnerability to develop negative health outcomes (Chye et al., 2018; Wei et al., 2018; Yuan et al., 2021). The metabolic alterations that underlie frailty can exacerbate other clinical conditions in the elderly, so it is necessary to carry out more studies in this regard.

In sum, the use of PBMC as an accessible biomarker and the measurement of OCR as a reflection of mitochondrial respiratory capacity may be valuable tools to identify initial stages of functional decline in older adults, such as frailty.

## Data Availability

The raw data supporting the conclusion of this article will be made available by the authors, without undue reservation.

## Supplementary material

SUPPLEMENTARY FIGURE S1 Representative plot of oxygen consumption rate (OCR) measurement. Before the addition of the cells into the chamber of the oxygraph, the oxygen concentration is at baseline level. After addition of the cells there is a stabilization window characterized by spikes, which correspond to the pipetting of the cells and the closing of the chamber. After the chamber is closed, the oxygen concentration within the chamber steadily decreases and the slope of the decrease corresponds to the OCR.
