# Supplementary figures and images for: Decreased mitochondrial respiration associates with frailty in community-dwelling older adults
Source: Front Cell Dev Biol. 2024 May 7;12:1301433. doi: 10.3389/fcell.2024.1301433 (PMC11110568; doi:10.3389/fcell.2024.1301433)

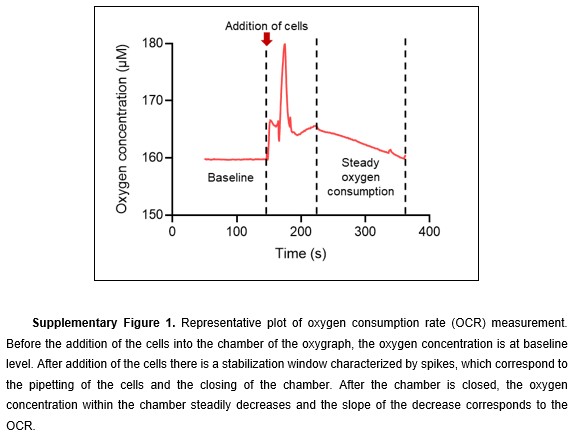

Supplement: Supplementary file 1 [file Image1.jpg]
